# Population Vulnerability and Disability in Kenya's Tsetse Fly Habitats

**DOI:** 10.1371/journal.pntd.0000957

**Published:** 2011-02-08

**Authors:** Sue C. Grady, Joseph P. Messina, Paul F. McCord

**Affiliations:** 1 Department of Geography, Michigan State University, East Lansing, Michigan, United States of America; 2 Department of Geography, Center for Global Change and Earth Observation, Michigan Agricultural Experiment Station, Michigan State University, East Lansing, Michigan, United States of America; 3 Department of Geography, Center for Global Change and Earth Observation, Michigan State University, East Lansing, Michigan, United States of America; Foundation for Innovative New Diagnostics (FIND), Switzerland

## Abstract

**Background:**

Human African Trypanosomiasis (HAT), also referred to as sleeping sickness, and African Animal Trypanosomaisis (AAT), known as nagana, are highly prevalent parasitic vector-borne diseases in sub-Saharan Africa. Humans acquire trypanosomiasis following the bite of a tsetse fly infected with the protozoa *Trypanosoma brucei (T.b.) spp.* –i.e., *T.b. gambiense* in West and Central Africa and *T.b. rhodesiense* in East and Southern Africa. Over the last decade HAT diagnostic capacity to estimate HAT prevalence has improved in active case-finding areas but enhanced passive surveillance programs are still lacking in much of rural sub-Saharan Africa.

**Methodology/Principal Findings:**

This retrospective-cross-sectional study examined the use of national census data (1999) to estimate population vulnerability and disability in Kenya's 7 tsetse belts to assess the potential of HAT-acquired infection in those areas. A multilevel study design estimated the likelihood of disability in individuals, nested within households, nested within tsetse fly habitats of varying levels of poverty. Residents and recent migrants of working age were studied. Tsetse fly's impact on disability was conceptualised via two exposure pathways: directly from the bite of a pathogenic tsetse fly resulting in HAT infection or indirectly, as the potential for AAT takes land out of agricultural production and diseased livestock leads to livestock morbidity and mortality, contributing to nutritional deficiencies and poverty. Tsetse belts that were significantly associated with increased disability prevalence were identified and the direct and indirect exposure pathways were evaluated.

**Conclusions/Significance:**

Incorporating reports on disability from the national census is a promising surveillance tool that may enhance future HAT surveillance programs in sub-Saharan Africa. The combined burdens of HAT and AAT and the opportunity costs of agricultural production in AAT areas are likely contributors to disability within tsetse-infested areas. Future research will assess changes in the spatial relationships between high tsetse infestation and human disability following the release of the Kenya 2009 census at the local level.

## Author Summary

The tsetse fly's influence on human health occurs through direct and indirect exposure pathways. Directly, the fly is a vector for the disease human African trypanosomiasis (HAT), which it spreads to nearly 18,000 new victims each year. Indirectly, the fly is a vector for African Animal Trypanosomaisis (AAT) also known as nagana, which restricts agricultural production, limiting the availability of food and contributing to impoverished conditions across rural sub-Saharan Africa. This historical study used 1999 census data to determine the prevalence of disability among residents and migrants living within Kenya's 7 tsetse fly belts. The results showed that the HAT transmission cycle may differ for residents and migrants with mechanisms leading to exposures that are environmentally driven for residents and economically driven for migrants. The combined burdens of HAT and AAT and the opportunity costs of agricultural production in AAT areas are potential contributors to disability within these tsetse-infested areas. Incorporating reports on disability from the national census appears to be an important surveillance tool that would enhance future HAT surveillance programs in sub-Saharan Africa.

## Introduction

Human African Trypanosomiasis (HAT), also referred to as sleeping sickness, and African Animal Trypanosomaisis (AAT), known as nagana, are highly prevalent parasitic vector-borne diseases in sub-Saharan Africa. Humans acquire trypanosomiasis following the bite of a tsetse fly infected with the protozoa *Trypanosoma brucei (T.b.) spp.* –i.e., *T.b. gambiense* in West and Central Africa and *T.b. rhodesiense* in East and Southern Africa. AAT is spread by tsetse flies carrying a variety of trypanosomes, including *T. vivax*, *T. congolense*, *T.b. brucei*, and *T. simiae*. Tsetse flies belong to the genus *Glossina* and are divisible into three groups: *G. morsitans*, *G. palpalis* and *G. fusca*. Tsetse species are k-strategists and thrive in biome-specific environments defined by climate (temperature, soil moisture), vegetation and fauna [1].

In 1995, 36 sub-Saharan African countries reported approximately 40,000 new cases of HAT through passive surveillance but it was estimated that 300,000 to 500,000 additional cases remained undiagnosed and therefore, untreated [2]. If left untreated, HAT is fatal [3]. In 2000, the World Health Organization along with public-private partnerships initiated programs to enhance HAT surveillance in sub-Saharan Africa that had suitable habitats for tsetse flies [3]. Active surveillance programs were implemented in 24 countries endemic for *T.b. gambiense* and 13 countries endemic for *T.b. rhodesiense* [4] (Uganda is counted twice because of the overlap of agents). In 2004, the number of new cases dropped to 17,500, cumulative incidence 50,000 to 70,000 cases [4]. Of these, 17,036 cases were reported in *T.b. gambiense* endemic countries, with 89.6% of cases from Democratic Republic of the Congo, Congo, Angola and Sudan [4]. In *T.b. rhodesiense* endemic countries 580 new cases were reported in 2004, with 81.8% of cases from Uganda and United Republic of Tanzania [4]. This dramatic decline in incidence during the period of active surveillance was attributed to increased awareness and subsequent participation in prevention and control activities.

Much of rural sub-Saharan Africa however, is still without HAT-diagnostic capacity and passive surveillance programs to estimate population prevalence outside of active case-finding areas. For example, in 2007 Médecins sans Frontiérs (MSF), an international nongovernmental organization launched HAT control programs in rural villages in northern Democratic Republic of Congo that did not have active surveillance programs and found the population prevalence rate, 3.4% with 60% of cases in the first stage of disease, suggestive of intense transmission [5]. HAT control activities had not taken place in these villages for over three decades due to conflict in this region [5]. Other countries, such as Kenya that report fewer than 50 HAT cases per year, with the most recent confirmed case in March 2009 [6] have tsetse habitats in rural areas that support tsetse vectors for *T.b. rhodesiense* and also border HAT endemic countries -i.e., Uganda and United Republic of Tanzania that are also in need of enhanced HAT surveillance programs.

AAT also affects rural sub-Saharan Africa, and the effects of AAT most heavily impact sub-Saharan Africa's poor as 85% of these individuals live in rural areas, with over 80% relying on agriculture for their livelihoods [7]. AAT is responsible for over 3 million cattle and other livestock deaths each year across sub-Saharan Africa [8] with more than 46 million cattle at risk of contracting the disease [9] leading to a considerable impact on the agricultural economy. Direct production losses amount to approximately $1.2 billion each year [10]. Estimates rise to as much as $4.7 billion a year [11] when indirect losses from the inability to use land and livestock to their fullest potential, such as drawing on livestock for traction, are considered. Livestock productivity is necessary if poverty is to be reduced and health improved; livestock provide food (meat and milk), assist in crop production, and provide a source of income for some of the most marginalized rural citizens [12]. Moreover, if nutritional requirements are compromised in populations, morbidity and mortality from other types of infectious diseases increases [13]. Accordingly, AAT is a proximate contributor to poverty, food insecurity, and nutritional deficiencies in rural areas across sub-Saharan Africa.

**Figure 1:**
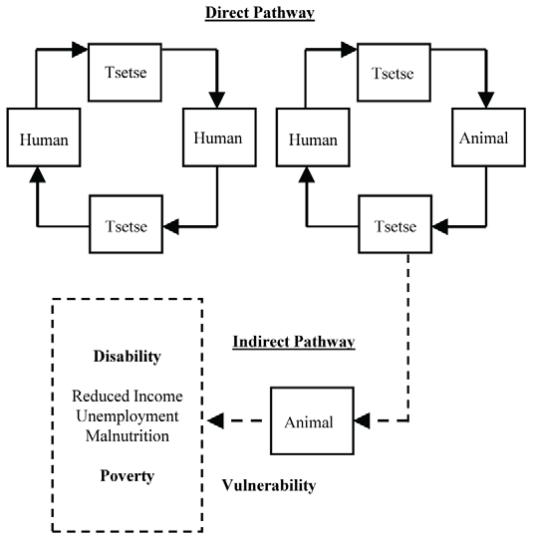
Conceptual model^1^ of *T.b. rhodesiense* sleeping sickness transmission cycle. ^1^Adapted from the World Health Organization, 2006.

The tsetse fly's impact on human health therefore occurs via two exposure pathways: directly from the bite of a pathogenic tsetse fly resulting in HAT infection or indirectly, as the potential for AAT takes land out of agricultural production and diseased livestock leads to livestock morbidity and mortality, contributing to poverty and nutritional deficiencies (Figure 1). Studies in East and West Africa [14], [15] report increased risk of HAT and AAT infection in foci of high tsetse infestation and close contact between tsetse fly, animal reservoirs and animal and human hosts. In areas with strong active surveillance the human health effects of trypanosomaisis may be controlled; however, in sub-Saharan Africa a large proportion of people live in rural areas that lack both adequate public health infrastructure to conduct passive HAT surveillance and health care facilities with diagnostic equipment and medical personnel to diagnose and treat HAT infection.

The purpose of this study is to examine the use of historic national census data as a tool to estimate population vulnerability and disability in Kenya's 7 tsetse belts, where tsetse vectors for *T.b. rhodesiense* and other trypanosoma agents that cause AAT were present. Individual level reports of disability collected during the Kenya National Census in 1999 and incorporated into the Integrated Public Use Microdata Series (IPUMS) census microdata for social and economic research [16] are utilized in this study. The definition of ‘disability’ used in the census falls under the umbrella of chronic diseases, and other common infectious diseases that may contribute to disabilities such as malaria, acquired immunodeficiency syndrome (AIDS) and HAT. This cross-sectional multilevel study estimates the likelihood of disability in individuals, nested within households, nested within tsetse fly habitats of varying levels of poverty. To model the direct exposure pathway, the effect of living in tsetse fly habitats on the likelihood of disability will be estimated controlling for individual and household level differences and area-level poverty. To model the indirect exposure pathway, the modifying effect of living in poverty and tsetse fly habitats on the likelihood of disability will be estimated controlling for individual and household level differences. Residents and recent migrants of working age –i.e., 15 to 64 years are analysed because of potential differences in duration of exposure to tsetse-endemic environments. Adults are studied excluding children and the elderly to minimize other potential causes of disability in these populations.

It is hypothesized that (a) the prevalence of disability will be higher in tsetse belts than outside tsetse belts, regardless of poverty levels; (b) the prevalence of disability will be higher among residents than migrants because of increased duration of direct-tsetse and indirect-tsetse/poverty exposure(s); and (c) the prevalence of disability for residents and migrants will vary by gender and housing characteristics. Gender differences may occur if peridomestic responsibilities contribute to variation in direct and indirect exposure(s). Housing type may offer varying levels of protection in tsetse-endemic environments. This approach of utilizing historical national census data to assess population vulnerability and disability within and across tsetse belt regions as a potential indicator of HAT-acquired infection has not been conducted in Kenya or other rural areas of sub-Saharan African countries and, therefore, warrants investigation.

**Figure 2:**
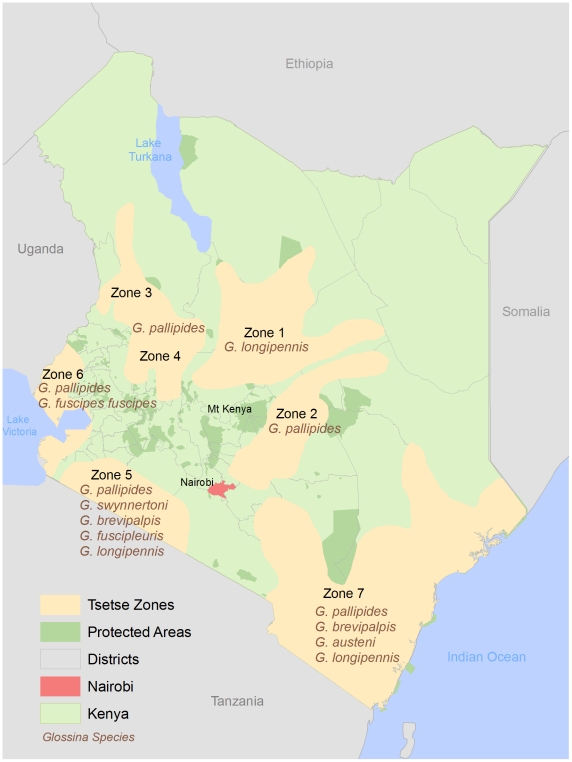
Tsetse habitats (Zones 1–7) and *Glossina spp.*, Kenya, 1999. Data Source: International Livestock Research Institute, Kenya 2009.

**Figure 3:**
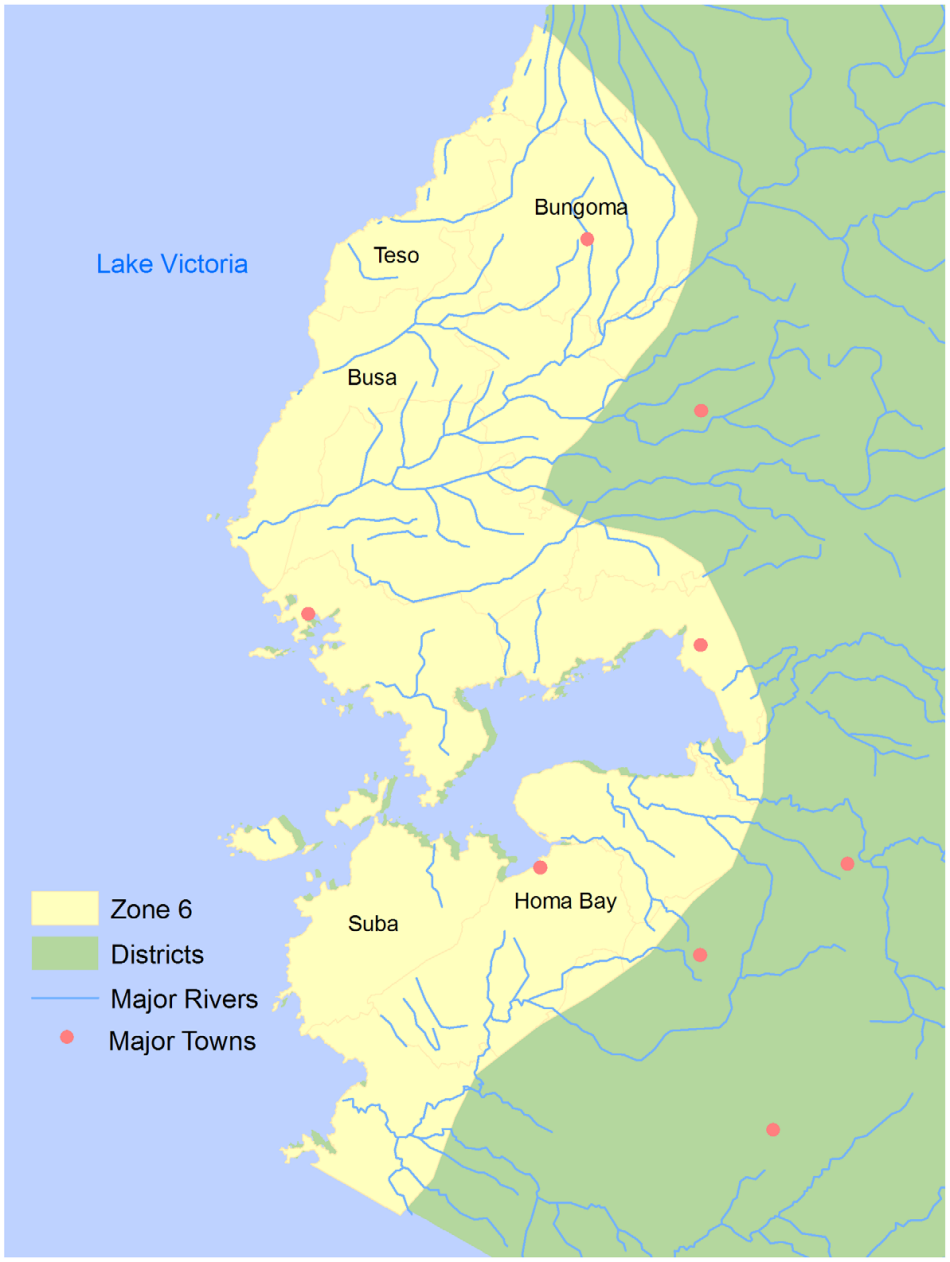
Tsetse belt (Zone 6) districts of HAT cases, Kenya 1999. Data Source: World Health Organization, 2007.

**Figure 4:**
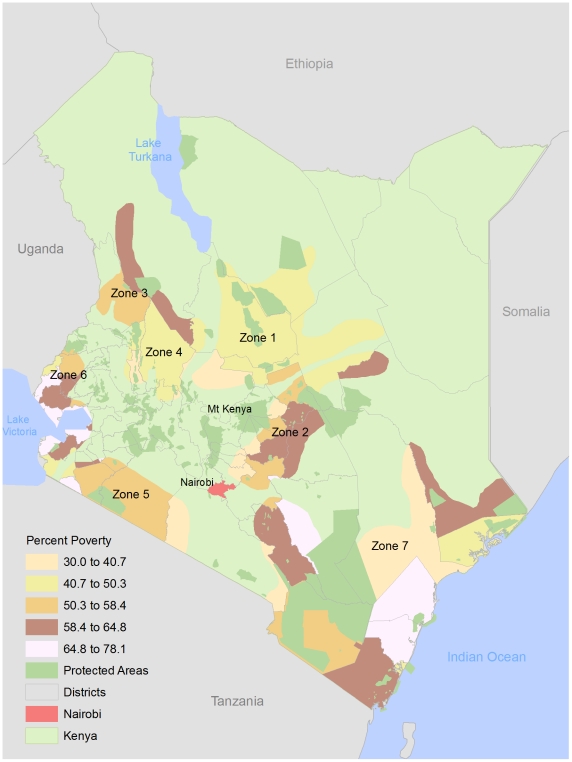
Percent of population living in poverty and tsetse habitats, Kenya, 1999. Data Source: International Livestock Research Institute, Kenya 1999.

Kenya is host to eight species of the tsetse fly [17] distributed across 7 tsetse belts, referred to as Zones 1–7 (Figure 2). Over the last century there have been 5 distinct HAT epidemics in Kenya, all of which were recorded in the Nyanza and Western Provinces along the Uganda border and shores of Lake Victoria (Zone 6 in this study) (Figure 3). The most recent epidemics were in 1964–1965 soon after independence, 1980–1984 in Nyanza Province and 1989–1990 in the Western Province [17]. HAT cases reported from 1977–1980 were relatively isolated in Busia and Teso, followed by an outbreak (n = 53) in Suba, Homa Bay and Migori that lasted until 1990 at which time no subsequent cases were reported in Nyanza Province [18]. The elimination of HAT infection in this area was attributed to tsetse control programs [18]. From 1986–1990 an outbreak occurred in Busia and Teso (n = 165) located south of the initial foci in the Western Province, with cases subsiding over time (n = 86) including sporadic cases in contiguous districts (n = 15) [18]. The total number of reported HAT cases in Zone 6 from 1950 to 2007 was 3,539 [18]. Since 2007 this foci has remained active with isolated HAT cases reported in villages in the districts of Bungoma, Busia and Teso [19]. The most recent reported case in March 2009 did not have a travel history indicating that transmission was local [6]. Importantly, no HAT cases have been reported in any of the other tsetse belts in Kenya despite the presence of capable tsetse vectors for *T.b. rhodesiense* in Zones 2, 3–4, 5, 6 and 7. There are also tsetse vectors for trypanosoma agents that cause AAT in cattle and livestock in Zones 1, 2, 3–4, 5, 6, and 7. In 1999, the proportion of the population living in poverty within the 7 tsetse belt zones is provided in Figure 4.

## Materials and Methods

### Data

This study is conducted at the district level (n = 69) in Kenya. The tsetse belt and district geography were obtained from the International Livestock Research Institute (ILRI) [20] for Kenya. Other district level data also obtained from ILRI included area level poverty (i.e., the percentage of the population living in poverty in each district). This dataset was joined to district geography and input into ArcGIS 9.3 [21] to calculate the proportion of the district that fell within each tsetse belt and this weight was used in subsequent analyses.

The Integrated Public Use Microdata Series (IPUMS) International dataset for Kenya managed by the Minnesota Population Center [16] was used to study disability in individuals. The IPUMS data were derived from the Kenya 1999 census collected by the National Bureau of Statistics [22]. The IPUMS data are a systematic sample of every twentieth household, which represented a sampling fraction of 5% and expansion factor equal to 20. A long form questionnaire was implemented surveying individuals within households. This sampling frame resulted in 28,150,940 men and women age 15 to 65 years (before expansion factor, n = 1,407,547) nested within 6,342,120 households (before expansion factor, n = 317,106) for use in this study. The data on disability and demographic and household characteristics came from this dataset. These data were examined and descriptive analyses were conducted in SPSS version 18 [23].

### Methods

The prevalence of disability was calculated for residents and migrants using the number of reported disabled individuals aged 15 to 64 years divided by the population 15 to 64 years * 1,000 (age-group-specific prevalence rates). Since the IPUMS data did not distinguish between new or existing cases of disability, prevalence was calculated rather than incidence. Disabled persons were identified as those who responded to the survey question, “What was (individual) mainly doing during the last 7 days preceding the Census night?” The response used to determine disability used in this analysis was “Unable to work, disabled” [16].

Multivariate multilevel analyses were conducted in Hierarchical Linear Modelling (HLM) software version 6.0 [24], [25]. Three-level hierarchical generalized linear model (HGLM) were implemented to estimate the variation in disability among individuals (level-1), nested within households (level-2), nested within weighted districts that comprised tsetse belts, districts outside tsetse belts and area-level poverty (level-3).

The dependent variable was ‘disability’ (Yes = 1). The level-1 characteristic of individuals used as an independent variable and the format of the data included gender (Female  = 1). The level-2 household dwelling characteristics included roof (Grass, Palm = 1), electricity (No = 1), piped water (No = 1), flush toilet (No = 1) and septic sewage (No = 1). At level-3 the tsetse belts were modelled as Zone 1 = 1, 0 = not Zone 1, Zone 2 = 1, 0 = not Zone 2, etc., the percent of the population living in poverty (continuous) and Zone1*poverty, Zone2*poverty, etc. All analyses were stratified by migration status. Residents were defined as those persons 15 to 64 years of age who lived in the same district at least one year prior to the interview. This population was studied because past research has shown that people of working age in rural areas are at increased risk of HAT because of outdoor exposure(s) to tsetse flies during agricultural-related activities [26], [27]. Migrants were defined as those persons 15 to 64 years who lived in a different district one year prior to their interview. For descriptive analyses migrants were further divided into movers -i.e., persons who moved from one district to another within the tsetse belt and immigrants -i.e., persons who emigrated from another district within Kenya or another country outside of Kenya.

The first statistical model was fully unconditional and was implemented to learn how much variation in disability was allocated at each of the three levels. The fully unconditional model showed the disability for each individual as a function of the household mean plus a random error:
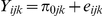
where, 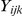
was the likelihood of disability for adult *i* in household *j* and tsetse belt *k*; 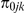 was the mean level of disability of household *j* in tsetse belt *k*; and 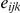
was the random “individual effect” -i.e., the deviation of individual *ijk*'s level of likelihood of disability from the household mean in tsetse belt *k*. These effects were assumed normally distributed with a mean of 0 and variance 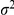. Herein, the indices *i*, *j*, and *k* denoted individuals, households and tsetse belts, respectively.

Each household mean 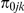, was an outcome varying randomly around some tsetse belt mean:
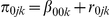
where, 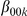
was the mean level of the household characteristic and 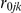 was the random “household effects” -i.e., the deviation of household *jk*'s mean from the tsetse belt mean. These effects were assumed normally distributed with a mean of 0 and variance 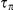. Within each of the *k* tsetse belts, the variability among households was assumed the same.

The level-3 model represented the variability among tsetse belts. The weighted district (herein referred to as district) and districts outside tsetse belts were viewed as the district means, 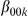
varying randomly around the grand mean: 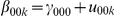
where, 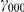
was the grand mean; 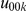
was a random “district effect” -i.e., the deviation in district *k*'s mean from the grand mean. These effects were assumed normally distributed with a mean of 0 and variance 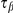. With this model the proportion of disability variation in individuals within households 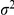 (level-1) was estimated; among households and within districts, 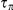 (level-2); and among districts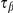 (level-3).

The conditional models allowed for the estimation of variability associated with the three levels. It was assumed that the variability at each level could be explained by the measured variables at each level. For example,

Level-1
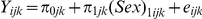
Level-2
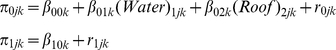
Level-3
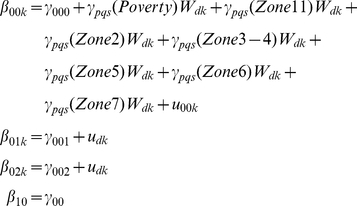



## Results

### Descriptive Analysis

The descriptive results from this study are provided in Tables 1–5. Table 1 shows that almost 50% of male and female residents and migrants worked in agriculture on family holdings *within* Kenya's tsetse belts. Residents worked in order of magnitude in Zones 1, 6, 7, 3–4, 5 and 2. Migrants worked in order of magnitude in Zones 1, 7, 6, 3–4, 5 and 2. One-third of migrants were movers (range, Zone 3–4, 18.91% to 31.53%, Zone 7) or immigrants from outside tsetse belts (range, Zone 3–4, 26.08% to 79.22% in Zone 5). The majority of international immigrants were from Uganda (Zone 3–4, 84.19% to 92.36%, Zone 6), Tanzania, Ethiopia, Somalia and other African countries not specified (data not shown).

**Table 1:** Residents and migrants(1) working in agriculture on family holdings within and outside tsetse belts, Kenya 1999.

	Residents				Migrants			
	Males		Females		Males		Females	
Tsetse Belts	No.	(%)	No.	(%)	No.	(%)	No.	(%)
Zone 1	283187	11.57	460338	13.69	14423	14.74	31004	20.65
Zone 2	71413	2.92	67337	2.00	1705	1.74	1950	1.30
Zone 3 & 4	114957	4.70	156985	4.67	6525	6.67	8410	5.60
Zone 5	93636	3.83	101394	3.01	2605	2.66	3070	2.04
Zone 6	220758	9.02	330320	9.82	6426	6.57	8618	5.74
Zone 7	203991	8.34	332432	9.88	7855	8.03	10560	7.03
Subtotal	987942	40.38	1448806	43.08	39539	40.40	63612	42.37

Outside Tsetse Belts								
Nairobi	13760	0.56	14540	0.43	2140	2.19	5380	3.58
Other Districts	1445018	59.06	1899754	56.49	56181	57.41	81148	54.05
Subtotal	1458778	59.62	1914294	56.92	58321	59.60	86528	57.63
Grand Total	2446720	100.00	3363100	100.00	97860	100.00	150140	100.00

(1) Residents are those living in same tsetse belt, district or Nairobi longer than 1 year; migrants are those living in tsetse belt, district or Nairobi less than 1 year; all residents and migrants are ages 15 to 64 years.

Source: Kenya National Bureau of Statistics; Minnesota Population Center. *Integrated Public Use Microdata Series-International: Version 5.0*. Minneapolis: University of Minnesota, 2009.

**Table 2 pntd-0000957-t002:** Household characteristics within and outside tsetse belts, Kenya 1999.

	Household	Grass	Dirt	No	No Flush	No Piped	No Septic
	Dwellings	Roofs	Floors	Electricity	Toilet	Water	Sewage
Tsetse Belts	No.	(%)	(%)	(%)	(%)	(%)	(%)
Zone 1	3136890	45.39	75.73	95.04	77.80	91.40	98.60
Zone 2	588590	50.28	85.41	96.14	94.30	77.36	97.40
Zones 3 & 4	1102385	53.59	85.77	96.66	97.22	94.90	98.14
Zone 5	810399	58.87	80.00	93.67	96.62	88.23	97.37
Zone 6	2219130	17.60	66.76	93.38	94.89	76.57	95.68
Zone 7	3184998	49.38	62.53	85.70	89.97	60.73	91.91

Outside Tsetse Belts							
Nairobi	2085820	0.02	12.73	41.04	37.87	9.01	40.96
Other	15022728	17.14	71.08	91.56	98.23	80.45	95.34
Total	28150940	26.02	67.10	88.04	90.12	74.56	91.32

Source: Kenya National Bureau of Statistics; Minnesota Population Center. *Integrated Public Use Microdata Series-International: Version 5.0*. Minneapolis: University of Minnesota, 2009.

**Table 3 pntd-0000957-t003:** Prevalence rates of disability for male and female residents(1) living within and outside tsetse belts, Kenya 1999.

	Disabled				Prevalence Rate(2)		
	Males		Females				
Tsetse Belts	No.	(%)	No.	(%)	Males	Females	Overall
Zone 1	4603	16.08	6436	19.15	7.16	8.15	7.70
Zone 2	540	1.89	488	1.45	3.88	3.38	3.63
Zones 3 & 4	821	2.87	1048	3.12	3.54	3.96	3.77
Zone 5	690	2.41	693	2.06	3.75	3.53	3.63
Zone 6	2516	8.79	2678	7.97	4.76	4.49	4.62
Zone 7	3276	11.45	3682	10.96	4.27	4.53	4.40
Subtotal	12446	43.49	15025	44.72	4.99	5.36	5.19

Outside Tsetse Belts							
Nairobi	1600	5.59	1160	3.45	2.35	2.27	2.31
Other Districts	14574	50.92	17415	51.83	4.14	4.57	4.36
Subtotal	16174	56.51	18575	55.28	3.85	4.30	4.08
Grand Total	28620	100.00	33600	100.00	–	–	–

(1) Residents are males and females 15-64 years living in same tsetse belt, district or Nairobi longer than 1 year.

(2) Includes residents who report “Unable to work, disabled” per 1,000 population 15-64 years at risk.

Source: Kenya National Bureau of Statistics; Minnesota Population Center. *Integrated Public Use Microdata Series-International: Version 5.0*. Minneapolis: University of Minnesota, 2009.

**Table 4 pntd-0000957-t004:** Prevalence rates of disability for male and female migrants(1) living within and outside tsetse belts, Kenya 1999.

	Disabled				Prevalence Rate(2)		
	Males		Females				
Tsetse Belts	No.	(%)	No.	(%)	Males	Females	Overall
Zone 1	250	10.50	535	17.83	4.08	6.55	5.49
Zone 2	24	1.01	13	0.43	2.55	1.63	2.12
Zones 3 & 4	67	2.82	55	1.83	3.30	2.83	3.07
Zone 5	101	4.24	157	5.23	6.61	12.19	9.16
Zone 6	139	5.84	105	3.50	4.01	3.47	3.76
Zone 7	277	11.64	325	10.83	2.41	3.38	2.85
Subtotal	858	36.05	1190	39.67	3.35	4.79	4.06

Outside Tsetse Belts							
Nairobi	420	17.65	320	10.67	2.14	1.61	1.87
Other Districts	1102	46.30	1490	49.67	2.17	3.28	2.69
Subtotal	1522	63.95	1810	60.33	2.16	2.77	2.45
Grand Total	2380	100.00	3000	100.00	–	–	–

(1) Migrants are males and females 15-64 years living in tsetse belts, districts or Nairobi less than 1 year.

(2) Includes migrants who report “Unable to work, disabled” per 1,000 population 15-64 years at risk.

Source: Kenya National Bureau of Statistics; Minnesota Population Center. *Integrated Public Use Microdata Series-International: Version 5.0*. Minneapolis: University of Minnesota, 2009.

**Table 5:** Multilevel model of disability in residents by tsetse habitat(1), Kenya 1999.

Fixed Effect	Coefficient	Odds Ratio	95% CI	p value
Intercept	-6.145	0.157	0.002, 0.003	0.000
Poverty	0.938	2.555	1.360, 4.800	0.005
Zone 1	0.464	1.591	1.249, 2.209	0.000
Zone 2	0.095	1.100	0.827, 1.463	0.506
Zone 3-4	-0.204	0.814	0.602, 1.102	0.181
Zone 5	0.013	1.013	0.773, 1.328	0.923
Zone 6	0.287	1.333	1.054, 1.687	0.018
Zone 7	0.122	1.130	0.848, 1.507	0.398
No Piped Water	0.045	1.046	1.032, 1.061	0.000
Female	-0.112	0.893	0.822, 1.061	0.009

(1) Levels 1 and 2 variables (sex and water) are modelled group centered;

Level 3 variables are modelled as natural metric.

(Model Estimating Potential Direct Exposures).

Table 2 shows that housing characteristics were relatively similar inside and outside tsetse belts except that a higher proportion of people living within tsetse belts had “grass roofs” (49.38%) compared to outside (17.14%) tsetse belts. Housing within Nairobi, the capital city of Kenya, was substantially different with fewer people living with grass roofs, dirt floors or no piped water.

Tables 3 and 4 provide the prevalence rates of disability for residents and migrants. Overall the prevalence of disability was higher within tsetse belts than outside tsetse belts for both residents and migrants; however, the within/outside rate ratio (RR) was greater for migrants (RR = 1.65) than residents (RR = 1.24). The within/outside RR for migrants was greatest in Zone 5 (RR = 3.73) and Zone 1 (RR = 2.24) and for residents the RR = 1.88 was greatest in Zone 1. The prevalence of disability was lowest in Nairobi for both residents and migrants. A description of the people, physical attributes and prevalence rates of disability in each of Kenya's 7 tsetse belts is provided below.

Zone 1, is predominantly arid and semiarid lands of desert shrub and grass savannah and is located north of Mt. Kenya, including parts of the Rift Valley and Eastern Provinces. Ethnically, the Nilo-Hamitic people of the Samburu tribe dominate, with the Hamitic Somali tribe occupying a small region in the south and the Hamitic Galla tribe in the eastern projection of the belt [3]. The area lies north of high potential agricultural lands and has traditionally been used as a rangeland [7]. Recently, there has been a conversion to commercial large-scale wheat production in a large part of the Samburu lands. Since 1967, an influx of people into traditional grazing lands resulted in shrubland deforestation and the destruction of tsetse habitat [8], resulting in a redistribution of tsetse. The presence of *G. longipennis* has been confirmed in the southwest corner of the belt [9], [10] and may be a reservoir for *Trypanosome brucei spp.* that infects domestic and wild animals. AAT is not often found in areas infested by *G. longipennis*, however, as this species thrives in tropical forests and/or forest outliers unfavourable to the tending of livestock. Zone 1 had the highest proportion of residents and migrants working in agriculture on family holdings, which may have been related to the rise of commercial wheat production. The prevalence of disability for residents was highest in Zone 1, 7.70 per 1,000 population and was second highest for migrants, 5.49. Of migrants, the prevalence of disability for movers was 5.42, which was less than that of immigrants from outside the tsetse belt, 6.00 and international immigrants, 8.29.

Zone 2, the Central Kenya belt, incorporates parts of the Central and Eastern Provinces and is mostly populated by Bantu people with Hamitic people in the northern projection of the belt [3]. This area includes parts of Kenya's productive highlands, which have a higher elevation and much moister climate. These areas have high agricultural potential and are intensely farmed [7]. *G. pallidipes* have been confirmed in Zone 2 [9], [10] but no HAT cases have been reported. Zone 2 had the lowest proportion of resident and migrant agricultural workers on family holdings, suggestive that the productive farmland was otherwise managed. Despite Zone 2′s high agricultural potential, a majority of the population lived in poverty. The overall prevalence of disability among residents in Zone 2 was 3.63 compared to migrants, 2.12. Of immigrants the prevalence for movers was 2.65 compared to 3.25 for international immigrants. There were too few immigrants from outside this tsetse belt to calculate a prevalence rate.

Zones 3–4 are geographically related and mainly inhabited by Nilo-Hamitic people of the Turkana tribe [3]. These rangeland zones comprise the North and South Rift Valley Belts, lie just east of Uganda within the Rift Valley Province and are longitudinally split by the Great Rift Valley. The climate ranges from dry sub-humid in the southern part of the belt to semi-arid in the north with dramatic wet and dry seasons [7]. Vegetation consists mainly of desert shrubs and grasses, with savanna bordering the region to the south, east, and west [12]. As of 1996, the most common species of tsetse confirmed in the belts was *G. pallidipes* [9], [10], although no HAT cases have been reported in this zone. *G. pallidipes* may also be a vector for *T. brucei*, *T. congolense* and *T. vivax* agents capable of infecting cattle. AAT infection rate among cattle in Zone 3–4 was 12.9% [15]. The overall prevalence of disability was relatively similar for residents, 3.77 and migrants, 3.07. Of migrants, the prevalence of disability for movers was greater, 2.89, than immigrants from outside the tsetse belt (0.59) and less than international immigrants, 5.60.

Zone 5, Transmara-Narok-Kajiado, lies within the southern part of the Rift Valley Province along the Tanzanian border. The area is inhabited by the Nilo-Hamitic Masai tribe [3] and includes the Masai Mara Game Reserve. The belt lies just south of the high potential agricultural lands and the climate is predominantly semi-arid [7]. Vegetation includes a mix of savanna in the west, desert shrubs and grasses, and some marshland in the east [12]. The confirmed tsetse species are *G. swynnertoni*, *G. brevipalpis*, *G. pallidipes*, *G. fuscipleuris*, and *G. longipennis* [9], [10], capable vectors of *T. rhodensiense* and other agents that cause AAT in cattle and livestock. Zone 5, had a small proportion of residents and migrant workers in agriculture on family holdings; however, the prevalence of disability for migrants was highest in Zone 5, (9.16), with the prevalence of disability for migrant females (12.19), almost twice that of migrant males, 6.61. Of migrants the prevalence of disability for movers, 3.68, was substantially lower than immigrants from other districts, 34.28. Zone 5 experienced the greatest number of immigrants from outside tsetse belts and the high prevalence of disability in this group most likely explains the large disparity for migrants living inside versus outside tsetse belts RR = 3.73. The prevalence of disability for international immigrants was very low, 0.73. The prevalence of disability among residents was substantially lower, 3.63, with female prevalence, 2.06, relatively similar to male prevalence, 2.41.

Zone 6, the Western Kenya and Lake Victoria belt, encompasses the Western and Nyanza provinces along the Ugandan border and the banks of Lake Victoria. The Nilotic people of the Luo tribe live along Lake Victoria and the Bantu Luhya tribe inhabits the northern part of the fly belt [12]. The climate is humid, and the region is part of the high potential agricultural zone and is thus used for intensive cropping [7]. Despite the high agricultural potential of this zone, the proportion of people living in poverty is extremely high –i.e., in some areas up to 75%. Tsetse species include widespread *G. fuscipes fuscipes and G. pallidipes* [9], [10]. *G. pallidipes* may host *T.b. rhodesiense* and HAT cases have been reported in this zone [18]. Both tsetse species are vectors for trypanosoma agents that cause AAT infection. The rate of infection among cattle was 8.3% [17]. The overall prevalence of disability for residents was slightly higher, 4.62, than for migrants, 3.76. Movers had a similar prevalence of disability, 1.69, as immigrants from outside the tsetse belt, 1.48, which was lower than for international immigrants, 5.81. Over 90% of international migrants emigrated from Uganda.

Zone 7, the Coastal belt, lies in the Coast Province along Kenya's eastern edge bordering 536 km of the Indian Ocean [13]. While the southwestern portion of the zone is ethnically diverse, the coast and Tana River region are dominated by Bantu people of the Mijikenda tribe [3]. Adjacent west of the zone are the Tsavo East and West National Parks and Chyulu Hills National Park. Vegetation transitions from mostly coastal brush, patches of coastal forest and mangrove swamp in the east to desert shrubs and grasses in the west, with riverine vegetation along the Tana River [12]. Tsetse species in this belt include *G. pallidipes*, *G. brevipalpis*, *G. austeni*, *and G. longipennis* [8], [9] capable vectors of *T. rhodesiense* and other trypanosoma agents responsible for AAT infection. The AAT infection rate among cattle was 15.6% [16], which was the highest of all tsetse belts. Zone 7 had the second highest proportion of residents and migrants working in agriculture on family holdings. The prevalence of disability for residents, 2.85, was less than that for migrants, 4.40. Movers had a slightly higher prevalence of disability, 3.95, than immigrants from outside the tsetse belt, 2.03, and international immigrants, 0.99.

### Analytical Results

The unconditional multilevel models showed that there was slightly greater variation in disability between residents 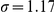 than between migrants (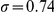). There was also significant variation in disability across tsetse belts and districts for residents (p value <0.005) but not migrants (p value >0.50), suggestive that migrant disability was more localized.

The conditional multilevel models provided information on risk factors for disability and these risks were compared for residents and migrants. Female residents were significantly less likely to be disabled than male residents (odds ratio (OR)  = 0.88, 95% CI, 0.81, 0.96) but there were not significant gender differences for migrants (p value <0.50). At the household level, not having piped water was the only significant risk factor for disability for both residents (OR = 1.03, 95% CI 1.00, 1.05) and migrants (OR = 1.06, 95% CI 1.02, 1.10), controlling for gender and other dwelling characteristics. Not having piped water, however, did not explain the geographic variability in disability for residents.

Table 5 shows the results from the conditional model that assesses the direct exposure pathway among residents -i.e., the effect of living in tsetse belts on disability. Two zones were highly significant, Zone 1 (OR = 1.59, 95% CI, 1.24, 2.20) and Zone 6 (OR = 1.33, 95% CI, 1.05, 1.68) controlling for gender, no piped water and area-level poverty. Living in these and other tsetse belts however, was not a significant risk factor for disability among migrants (data not shown).

Table 6 shows the results from the conditional model that assesses the indirect exposure pathway among residents -i.e., the estimated effect of living in poverty within tsetse belts. Three zones were highly significant for residents, in order of magnitude Zone 1 (OR = 2.66, 95% CI, 2.05, 3.48), Zone 6 (OR = 1.92, 95% CI, 1.38, 2.68) and Zone 7 (OR = 1.55, 95% CI, 1.10, 2.18) controlling for gender and no piped water. Interestingly, these same zones were also highly significant for disability in migrants, in order of magnitude Zone 1 (OR = 3.10, 95% CI, 2.16, 4.34), Zone 6 (OR = 2.21, 95% CI, 1.46, 3.37) and Zone 7 (OR = 1.72, 95% CI, 1.05, 1.07) (data not shown).

**Table 6:** Multilevel model of disability in residents by tsetse habitat and poverty(1), Kenya 1999 (model estimating potential indirect exposures).

Fixed Effect	Coefficient	Odds Ratio	95% CI	p value
Intercept	-5.667	0.003	0.003, 0.004	0.000
Zone 1*poverty	0.985	2.679	2.059, 3.489	0.000
Zone 2*poverty	0.329	1.390	0.895, 2.161	0.141
Zone 3-4*poverty	-0.033	0.967	0.674, 1.388	0.856
Zone 5*poverty	0.265	1.304	0.857, 1.985	0.211
Zone 6*poverty	0.656	1.928	1.387, 2.682	0.000
Zone 7*poverty	0.441	1.554	1.108, 2.182	0.012
No Piped Water	0.074	1.077	1.069, 10.86	0.000
Female	-0.091	0.912	0.852, 0.987	0.010

(1) Levels 1 and 2 variables (sex and water) are modelled group centered;

Level 3 variables are modelled as natural metric.

## Discussion

This retrospective cross-sectional study examined the use of historic national census data as a tool to estimate population vulnerability and disability in Kenya's 7 tsetse belts, where tsetse species are capable vectors for *T.b. rhodesiense* and other trypanosoma agents that cause AAT. This approach of utilizing national census data to assess population vulnerability and disability within and across tsetse belt regions, as a potential indicator of HAT-acquired infection has not been conducted in Kenya or other rural areas of sub-Saharan African countries and, therefore, warranted investigation. In 1999, Kenya's rural areas also lacked public health infrastructure for passive surveillance and rural health care facilities to adequately diagnose and treat trypanosomiasis infection [6], justifying the need for alternative surveillance approaches. It was hypothesized that (a) the prevalence of disability would be higher in tsetse belts than outside tsetse belts, regardless of poverty levels; (b) the prevalence of disability would be higher for residents than migrants because of increased duration of direct-tsetse and indirect-tsetse/poverty exposure(s); and (c) the prevalence of disability for residents and migrants would vary by gender and housing characteristics.

The results showed that residents living in Zones 1 and 6 were at increased odds of disability through the direct exposure pathway. Both residents and migrants living in Zones 1, 6 and 7 were at increased odds of disability through the indirect exposure pathway. Resident females were less likely to report disability than male residents and gender was not a significant risk factor for disability among migrants. Grass roofs were not a significant risk factor for disability despite there being a greater percentage of dwellings with grass roofs within tsetse belts than outside. Not having piped water, however, did increase the odds of disability for both residents and migrants, suggestive that agriculture and peridomestic activities, including the need to travel for potable water may increase exposure to tsetse or result in other disability-related morbidities. Lacking piped water did not explain the geographic variation in disability among residents.

To our knowledge Zone 1 does not contain tsetse species capable of transmitting *T. rhodesiense* thus HAT-related disability among residents due to direct tsetse exposure is unlikely. Furthermore, while *G. longipennis* was confirmed in the southwest corner of Zone 1 this tsetse fly does not thrive outside of tropical forests, thus HAT-related disability due to indirect exposure –i.e., poverty/lack of nutrition resulting from AAT is also unlikely. This unusual finding may be a limitation in the sampling of tsetse following their redistribution due to land cover and land use changes in Zone 1 –i.e., the conversion of traditional grazing land into commercial agriculture. Future research should therefore re-evaluate tsetse habitat and species distribution within Zone 1 in addition to potential change in the boundary definition. Future research should also explore other factors that may be associated with the significantly high rates of disability among agricultural residents and migrant workers on family land holdings in Zone 1. These factors may include changes in working conditions as a result of commercial agriculture dominating this region and/or the influx of migrants working in agriculture whose source of disability originated elsewhere.

Zone 6, however, is a HAT endemic area within which there is high potential for residents to have direct contact with pathogenic tsetse species. Zones 6 and 7 also have high rates of AAT infection in cattle, with a high proportion of the population residing in these zones also living in poverty. These findings support the direct exposure pathway to explain the significantly high prevalence of disability among residents and migrants in Zone 6 and the indirect exposure pathway to explain the significantly high prevalence of disability among residents and migrants in Zone 7.

Despite the presence of tsetse vectors for *T.b. rhodesiense* and other trypanosoma agents for AAT in Zones 6 and 7, it is unlikely that HAT is a primary driver of disability as measured here. Anecdotal support for HAT presence notwithstanding, the lack of medical facilities incapable of testing for the trypanosomes makes a statement of HAT presence presumptuous. However, AAT is present across these tsetse belts and the nutritional burden of diseased livestock is well documented [9]. It is expected that these nutritional burdens would also contribute to disability among the individuals found in tsetse belts, and, combined with the costs of treating diseased cattle, potentially manifest in the level of rural poverty.

In Kenya residents may be at increased odds of disability because of cumulative (also referred to as repeated) exposure to tsetse flies through various activities associated with agriculture and peridomestic chores. Repeated exposures to tsetse flies may be necessary to acquire infection if the seroprevalence of *T.b. rhodesiense* is low in *G. pallidipes* and *G. swynnertoni* species. Residents will have less ability to change locations of farming, raising livestock, hunting and fishing, and conducting peridomestic activities because of the permanency of their households and the financial response to AAT. In many areas, cattle are capital and the ability to accumulate liquid financial resources is severely constrained, thus enforcing a capital and nutritional feedback loop. High levels of poverty, resulting from constrained agricultural production, may also contribute to disability through food shortages and malnutrition. Protein-energy malnutrition and micronutrient deficiency may, put an individual at greater risk of HAT as such deficiencies increase morbidity and mortality from communicable diseases [13].

It is important to understand that the tsetse belts are areas of reported endemic tsetse, but should not be misconstrued as continuous exposure. It is likely that movers who change residency across districts in the same tsetse belts may have more choices of where to relocate, and in their decision making process there is a choice of whether or not to reside in active tsetse endemic areas. Therefore, the high prevalence of disability among movers is more likely explained by other morbidities such as malaria, or illnesses resulting from nutrient deficiency.

Immigrants from other districts within Kenya may be a part of the agricultural workforce and enter tsetse belt areas out of economic necessity. The land available for occupation in these cases is often the most infested with tsetse. This group overall had the highest prevalence of disability. Finally, migrants who emigrate from other countries, especially Uganda, also have a high prevalence of disability, suggesting that they acquired the disability elsewhere, such as their country of origin. Additionally, poverty is significantly associated with their disabilities, but it is likely that disabled emigrants immigrated into poor tsetse belt areas –e.g., Zones 6 and 7 rather than having acquired their disability within a year of their arrival. In Zone 6, 92.36% of international immigrants were from Uganda. Over 40% of Uganda is infested with tsetse flies with 70% of livestock grazing under risk of trypanosomiasis [28] leading to similar limitations on livestock and agricultural production as those experienced by Kenyans. Additionally, Uganda is an epicentre for both *T.b. gambiense* in the northwest and *T.b. rhodesiense* in the southeast portions of the country. Today these two foci have merged in north-central Uganda following political and social unrest, population displacement and livestock movement [27].

In East Africa and Kenya HAT acquired from *T.b. rhodesiense* infection is considered an acute form of the disease because of the rapid progression of disease (4 to 6 months) from the haemolymphatic stage (Stage 1) to the meningeoencephalitic stage (Stage 2). In Stage 1 the parasites invade the blood stream, lymphatic system and body tissues resulting in signs and symptoms of rash (trypanosomal chancre), fever, headache, enlarged cervical lymph nodes, edema, splenomegaly, hepatomegaly, and/or weight loss. If no treatment is initiated the disease will progress to Stage 2 as parasites cross the blood-brain barrier and invade the cerebrospinal fluid and the brain parenchyma [28], [29]. Signs and symptoms during Stage 2 reflect nervous system involvement, including fatigue, confusion, endocrine dysfunction, mental changes, poor coordination, sleep disturbances, and, if left untreated, lapse into coma and eventual death [28], [29]. The identification of the clinical stage of the disease is essential for proper drug treatment. No antibody-detecting test is available for screening populations for *T.b. rhodesiense*. The diagnosis of *T.b. rhodesiense* is made through parasitology of blood and chancres in Stage 1, and the examination of spinal fluid for parasites and white blood cells in Stage 2 [30]. New methods of serological testing for the detection and stage differentiation of *T.b. rhodesiense* and *T.b. gambiense* in poor rural areas of sub-Saharan Africa, including Kenya [6] are underway and will offer new capabilities for improved diagnosis and treatment of HAT in addition to improving the estimation of population prevalence [30]. The drugs that are used to treat *T.b. rhodesiense* infection include suramin in Stage 1 and melarsoprol in Stage 2 [31], [32]. Suramin does not cross the blood brain barrier and is therefore, prescribed in Stage 1. Melarsoprol does cross the blood brain barrier but is highly toxic (arsenic-based) and side effects are common [32]. Melarsoprol also has a high treatment-fatality rate with approximately 3–10% of all treated patients developing arsenical encephalopathy and death [31]. Melarsoprol, previously administered over a period of one month or more, can now be given over a 10-day period thereby reducing hospital costs [33], [34]. Minimizing the risk of treatment is, therefore, dependent upon an accurate diagnosis of the clinical stage of HAT infection.

The role of AAT in generating impoverished conditions and limiting food supplies cannot be overlooked in its influence on human health. The disease creates areas where livestock are virtually excluded, areas where only certain breeds resistant to trypanosomiasis can live, and/or areas where breeds susceptible to the disease can be raised due only to the confinement of tsetse habitat or the use of preventative treatments [35]. Each scenario leads to an underproduction in agricultural and livestock output. Livestock production is an important component of Kenya's economy at both the local and national level. It accounts for 12% of the country's total GDP and 47% of agricultural GDP [36], and is relied upon for much of the country's employment.

AAT has been shown to contribute to lower calving rates, lower milk and meat yields, higher rates of livestock mortality, and more frequent treatment with trypanocidal drugs. In fact, in tsetse-infested areas, trypanosomiasis reduces milk and meat offtake by at least 50% [8]. The largest indirect consequence of AAT is its ability to exclude animals that would provide animal traction in agricultural operations. Kristjanson et al. (2004) demonstrated the role of unproductive land and loss of livestock in generating poverty [37]. In their study, poverty in 10 communities of Kenya's Western Province and 10 communities of Kenya's Nyanza Province was assessed and revealed that 38% of households considered unproductive land as a factor in becoming poor. Additionally, 25% of households listed loss of livestock as a contributor to becoming poor. Poor health was listed most frequently as a major reason for becoming poor, suggesting that improved health care access needs to be a central priority in impoverished areas. Of those who had escaped poverty, 57% considered income from crop farming as a reason for their improved economic standing and 42% believed that diversification into livestock farming allowed them to escape poverty [37]. Thus, future efforts to reduce area-wide disability need to focus attention on making health facilities more accessible, as well as improving agricultural capabilities. This can only be accomplished through effective control of AAT and the tsetse fly.

The limitations of this study include: the census data used in this study are from 1999 so this historical perspective may not be the situation prevailing today, especially in relation to access to health care. In terms of methodology at level-3 the tsetse belts represented areas where tsetse flies had been identified and these areas were generalized to represent a “belt”. There may be, however, substantial spatial variation within the belt(s) not accounted for in this analysis [38]. At level-2 we did not distinguish between characteristics of household dwellings for residents and migrants. Migrants may reside in less well-maintained housing than residents who are permanently situated. Finally, at level-1 there may be other physiological characteristics and cultural and behavioural-related factors associated with the many tribes in rural Kenya that would help to explain the variation in prevalence of disability within and across tsetse belts and districts. Such factors may include nutritional and immunological status or nomadic status of tribal cultures. The tribal-cultural practices of Kenya's residents and immigrants and their behavior's pertaining to disability risk should be evaluated for future education and prevention programs.

Future research should determine how climate, land use and land change affect tsetse habitats, the vector-agent relationship and human interactions contributing to HAT and AAT infection to inform public health prevention and control activities. Mapping these relationships at a finer scale will assist in these efforts. In addition, a more detailed investigation into the factors contributing to rural poverty is warranted, particularly an investigation into the association of poverty and the tsetse fly at the local level. Such a study would compliment the work of Okwi et al [39] on poverty at the province level in rural Kenya. Finally, understanding the contribution of the nutritional and poverty related effects of AAT on other debilitating diseases such as malaria and HIV/AIDS in sub-Saharan Africa will also help to target populations and interventions that will further reduce the complexity of multiple disease burdens in sub-Saharan Africa.

## Supporting Information

Supporting Checklist S1 STROBE Checklist
(0.09 MB DOC)
